# P40 - Double-blind, placebo-controlled, food challenges (DBPCFC) of a strong tasting food: lessons learned

**DOI:** 10.1186/2045-7022-4-S1-P95

**Published:** 2014-02-28

**Authors:** Marlene Borschel, Jr A Wesley Burks

**Affiliations:** 1 Abbott Nutrition, Abbott Laboratories, Columbus, OH, USA; 2 University of North Carolina, Chapel Hill, NC, USA

## Background

Recommendations/regulations dictate the testing of hypoallergenic formula in allergic patients prior to marketing. The purpose of this study was to generate DBPCFC data on a casein hydrolysate-based infant formula (EF). It also provided information useful for conduct of DBPCFC of strong tasting foods.

## Methods

9 children (1.1-4.7 yr) with documented IgE-mediated cow milk (CM) allergy participated. DBPCFC were performed over a 3-d period in hospital using procedures described by Sampson et al (J Pediatr;118:520). A DBPCFC was performed on each of the first 2 d. Day 3, an open challenge of EF was done. Challenges were 100 mL of placebo (P) formula (Nutramigen^®^ liquid, Mead Johnson Nutritionals, Evansville, IN) or EF (Nutramigen containing 8 g freeze-dried Similac^®^ Alimentum^®^, Abbott Nutrition (AN), Columbus, OH). Flavoring (chocolate or strawberry syrup) was optional and, if chosen, used in all challenges.

## Results

At entry 6 subjects received a challenge to CM with positive results. The amount of CM protein eliciting a response ranged from 0.063-1.54 g. 3 subjects had a repeat reaction to CM due to accidental exposure just prior to entry and were not re-challenged. 8 subjects successfully completed the DBPCFC and open feeding of EF; 1 with a history of anaphylaxis to CM reacted to P and EF (both with chocolate syrup). 3 mo later, the subject returned for repeated DBPCFC because the syrup was suspected. The subject experienced a reaction to EF with strawberry syrup and was not challenged with P. These challenges were judged to be “inconclusive” because it was impossible to ascertain if the child experienced a reaction to P, EF, and/or the syrup(s).

## Conclusion and next steps

These data, along with those from Sampson et al (1991), were used to support current recommendations for hypoallergenic labeling for EF to document that with 95% confidence, EF was tolerated by at least 90% of individuals documented to be allergic to cow milk (CM) protein. It is recommended that challenge mixtures not include other foods or flavorings that have the potential to elicit allergic reaction individually.

